# The impact of work concerns on teaching effectiveness: a structural equation modeling and multiple regression analysis in Chinese private universities

**DOI:** 10.3389/fpsyg.2025.1661379

**Published:** 2025-09-18

**Authors:** Mingyu Liang, Mohd Khairuddin Abdullah, Qun Wang

**Affiliations:** ^1^School of Arts and Media, Qingdao Hengxing University of Science and Technology, Qingdao, China; ^2^Faculty of Education and Sport Studies, University Malaysia Sabah, Kota Kinabalu, Malaysia

**Keywords:** work concerns, teaching effectiveness, young teachers, structural equation modeling, multiple regression

## Abstract

It is crucial to understand how young teachers cope with work concerns for improving teaching quality in the Chinese private higher education institutions. This study investigates the relationship between different stages of such concerns and teacher effectiveness among young lecturers in private universities. These lecturers often face workload pressure and a lack of career support, which may influence their professional development and teacher effectiveness. This research involved 416 full-time lecturers under 40 from four private universities located in Shandong Province. The sample was selected through a multi-stage sampling method: private universities were stratified into four categories, one university from each category was purposively selected, and participants were randomly sampled. Data were collected using a structured questionnaire based on the Stages of Concern and the School Teacher Effectiveness Questionnaire. Pearson correlation, multiple regression, and structural equation modeling were conducted for analysis. Results show that task concerns and impact concerns significantly influenced teacher effectiveness across three dimensions: instructional planning and strategies, assessment, and learning environment. In contrast, self-concerns showed weaker influence. These findings suggest that work concerns reflect not only stress but also deeper professional motivation, pointing to the need for more targeted support strategies to improve teacher effectiveness and career growth.

## 1 Introduction

Chinese private universities have emerged as critical players in expanding access to tertiary education and promoting regional educational equity in the context of China’s higher education reforms (Li et al., 2022). According to the Ministry of Education in 2023, there are approximately 750 private institutions now serve more than six million students across the country. These institutions operate under distinct structural and policy environments, facing constraints such as limited public funding, weaker research capacity, and faculty recruitment challenges (Wang, 2022). In recent years, researchers have paid much more attention to the professional development and teaching effectiveness of young university teachers, as their work plays a crucial role in shaping both the quality of education and the reputation of their institutions (Liu et al., 2024; Xiao, 2023).

Young lecturers working in private universities are often in the early stages of their careers, facing the pressures of heavy workloads and limited professional support (Zhou and Wang, 2025). In Shandong province, where private higher education has expanded rapidly, issues such as high faculty turnover, a shortage of training opportunities, and limited access to research platforms are frequently reported (Bing, 2023). These conditions may lead to reduced teaching effectiveness. Although there have been policy efforts aimed at improving teaching standards in private institutions, there is still a gap in research on how young lecturers experience and manage their professional concerns in their daily work.

It’s still unclear whether the Concerns-Based Adoption Model (CBAM) developed by Fuller (1969) and Hall and Hord (2020) fits the Chinese universities. According to this model, teachers’ work concerns include different stages: self-concerns (focusing on themselves), task concerns (handling teaching tasks), and impact concerns (focusing on their impact on students). These different concerns are believed to shape how open when teachers use teaching methods and how active in their professional growth. Although CBAM has been widely used in K-12 schools in Western countries, it is still not clear whether this model applies to young lecturers working in China’s private universities (Fischer et al., 2019; George et al., 2008). Another key problem is teaching effectiveness which is crucial in students achievement and outcome (Engida et al., 2024). It reflects a teacher’s capacity to design proper teaching plans, implement effective instructional practices, monitor student learning, and maintain a positive classroom climate (Akram et al., 2022). As a multidimensional construct, it consists of various aspects of teaching performance, such as planning, instruction, classroom management, and assessment (Sabharwal and Miah, 2024). In this study, teaching effectiveness is conceptualized through three core dimensions: instructional planning and strategies, assessment, and learning environment.

Recent studies indicate that higher levels of work engagements are positively correlated with instructional behaviors, reflective teaching, and greater student achievement (Christopher and Newman, 2022; Walugembe et al., 2022). Mendez et al. (2024) found that teachers’ concerns on work can influence their psychological wellbeing and their instructional planning and classroom effectiveness. Nevertheless, few empirical studies have confirmed the predictive relationship between different stages of work concern and teaching effectiveness, especially in Chinese private universities, where teaching demands are very high, but support systems remain underdeveloped.

This study aims to fill that gap by revealing the relationship between work concerns and teaching effectiveness among early-career lecturers in Chinese private universities, using validated instruments such as the stages of concern questionnaire (SoCQ) and the school teacher effectiveness questionnaire (STEQ). This investigation focuses on three dimensions of teaching effectiveness: instructional planning and strategies, assessment, and learning environment. It also considers how different stage of concerns contribute to these outcomes. By using structural equation modeling (SEM) and multiple regression, this study provides a quantitative examination of how internal psychological concerns of young teachers shape their teaching effectiveness.

The contribution of this research lies in its use of the CBAM framework to the unique setting of Chinese private higher education and new empirical evidence to the global conversation on teacher development. Moreover, it offers guidance for institutional leaders who can help to in there management practice, such as mentorship, workload adjustment, and professional training, which could make young teachers more excellent. Therefore, this research addresses an important but often overlooked issue: how early-career teachers in Chinese private universities develop professionally. It brings both new ideas and practical advice to the field.

### 1.1 Objectives of the study

i) To check the current situation of work concerns and teacher effectiveness among young teachers in Chinese private universities.
ii) To investigate the relationships between work concerns and teaching effectiveness among young teachers in Chinese private universities.
iii) To examine the influence of work concerns on teaching effectiveness among young teachers in Chinese private universities.

### 1.2 Research questions

i) What is the stage of work concerns and the level of teacher effectiveness among young teachers in Chinese private universities?
ii) What is the relationship between work concerns and teacher effectiveness among young teachers in Chinese private universities?
iii) How do work concerns impact teacher effectiveness among young teachers in Chinese private universities?

## 2 Materials and methods

### 2.1 Research design

This research follows a quantitative approach, utilizing a cross-sectional survey to collect data. To examine the connections between various stages of work concerns and teaching effectiveness, structural equation modeling (SEM) is used. Additionally, multiple linear regression analysis is conducted to evaluate how well different stages of work concern can predict teaching effectiveness.

### 2.2 Population and sample

The population in this study comprised young full-time teachers employed in private universities across Shandong Province, China. The Chinese Ministry of Education defines young university lectures as those under the age of 40 or those who have <10 years of teaching experience with a master’s degree or higher. Shandong is one of China’s largest higher education hubs. Private universities in Shandong have expanded rapidly in the last two decades. However, young teachers here often face some challenges such as limited teaching experience, difficulty in adapting to digital teaching environments, and insufficient leadership and organizational support. These challenges result in uneven levels of teaching effectiveness and affecting student learning outcomes. Therefore, this study choose young lectures in Shandong’s private universities as research population.

According to the Statistical Bulletin on the Development of Education in Shandong Province (2023) issued by the provincial Department of Education, there are currently 44 private regular colleges and universities in the province, employing approximately 22,500 young teachers in 2024. It reflects a hiring trend that favors recent university graduates and early-career educators. These young teachers play a critical role in the development of private universities, which makes them especially important when studying teaching effectiveness and the challenges they face in their work (Ye et al., 2019). Their struggles and opportunities for career growth help to reveal key aspects in Chinese private universities. To make sure the sample was diverse, the study used stratified random sampling across university types (Howell et al., 2020). According to the sample size determination table by Krejcie and Morgan (1970), this study needs at least 380 participants.

To ensure the representativeness of the sample, the study employed a multistage sampling strategy. All 44 private universities in Shandong Province were first stratified into four categories: regular undergraduate, vocational undergraduate, independent colleges, and vocational colleges. From each category, one medium-sized university was purposively selected on the basis of typical institutional characteristics such as size, location, organizational structure, and the presence of young teaching staff. This approach ensured that the diversity of private universities in the province was adequately reflected. Within each of the four institutions, simple random sampling was then applied to select young lecturers, resulting in a total of 416 valid responses and an overall sampling ratio of 19.2%. Although purposive selection at the university level introduces some limitations, the overall strategy ensured that the sample was diverse, representative, and suitable for generalizing findings to similar educational contexts within China.

A total of 550 questionnaires were distributed across four private universities. Out of these, 450 completed questionnaires were returned, resulting in a response rate of 81.8%. After data screening procedures, 416 questionnaires were retained as valid responses, representing a valid response rate of 92.4% among returned surveys. Before collecting the data, participants were fully informed about the purpose of the study and were assured that their answers would be kept private. The study received ethical approval from all four participating universities, and all teachers provided informed consent.

### 2.3 Instruments

Data for this study were collected using a structured questionnaire with three sections, as shown in Table 1. The questions were adapted from two well-established sources: the stages of concerns questionnaire (SoC) by George et al. (2008) and the school teacher effectiveness questionnaire (STEQ) by Akram (2018). All items were rated on a five-point Likert scale, from 1 (strongly disagree) to 5 (strongly agree), based on how much participants agreed with each statement.

**Table 1:** Distribution of items.

Section	Dimensions	Items
Demographic details	Gender, age, teaching experience, qualification, and subject area	5
Work concerns	Self-concerns Task-concerns Impact-concerns	25
Teacher effectiveness	Instructional Planning and Strategies Assessment Learning environment	17

This questionnaire was adapted through a rigorous process to ensure validity in the Chinese private university context. The original instruments were reviewed by an educational management expert to evaluate content relevance and item clarity. Based on this review, ambiguous and redundant items were revised or removed. Then, the questionnaires were translated into Chinese by a bilingual specialist, and subsequently reviewed by a local educational management scholar to ensure cultural appropriateness and conceptual equivalence. To further strengthen validity, a back-translation into English was carried out by independent translators to verify consistency with the original versions. These procedures ensured that the items were linguistically accurate, culturally sensitive, and contextually meaningful.

#### 2.3.1 Section A: Demographic information

There are five items in this section to collect basic background information from the participants which covers age, gender, teaching experience, qualification, and academic subject area. Respondents simply needed to tick the box that matched their situation.

#### 2.3.2 Section B: Work concerns

This section was adapted form items in the stages of concerns questionnaire (SoC), which is widely used to assess teachers’ attitudes and concerns in their works. This study focus on three stages of work concerns among young teachers: self-concerns, which are about feeling confident and capable in their work; task-concerns, which deal with managing time and working duties; and impact-concerns, which reflect their desire in students’ learning achievements and outcomes.

#### 2.3.3 Section C: Teaching effectiveness

The final section was adapted from the school teacher effectiveness questionnaire (STEQ). In this research, the variable teaching effectiveness was measured using three key dimensions:

i) Instructional planning and strategies, focusing on how teachers design lessons and deliver content (Burt, 2022);
ii) Assessment, addressing how teachers evaluate student learning and use feedback (Black and Wiliam, 2018);
iii) Learning environment, concerning the classroom atmosphere and teacher-student interactions (Zoromski et al., 2021).

### 2.4 Pilot study

The questionnaire was initially reviewed by two experts in educational research to ensure clarity, cultural appropriateness, and alignment with the research objectives. A pilot test was also conducted with a small group of 288 participants. The results indicated that both work concerns and teaching effectiveness scales show high internal reliability, with Cronbach’s alpha coefficients ranging from 0.897 to 0.927, and all items showing strong item-total correlations. Exploratory factor analysis (EFA) identified three distinct factors for each scale, accounting for over 60% of the total variance, with all factor loadings exceeding 0.5. The KMO values (0.947 and 0.914, respectively) and significant Bartlett’s test results confirmed the suitability of the data for factor analysis.

**Figure 1:**
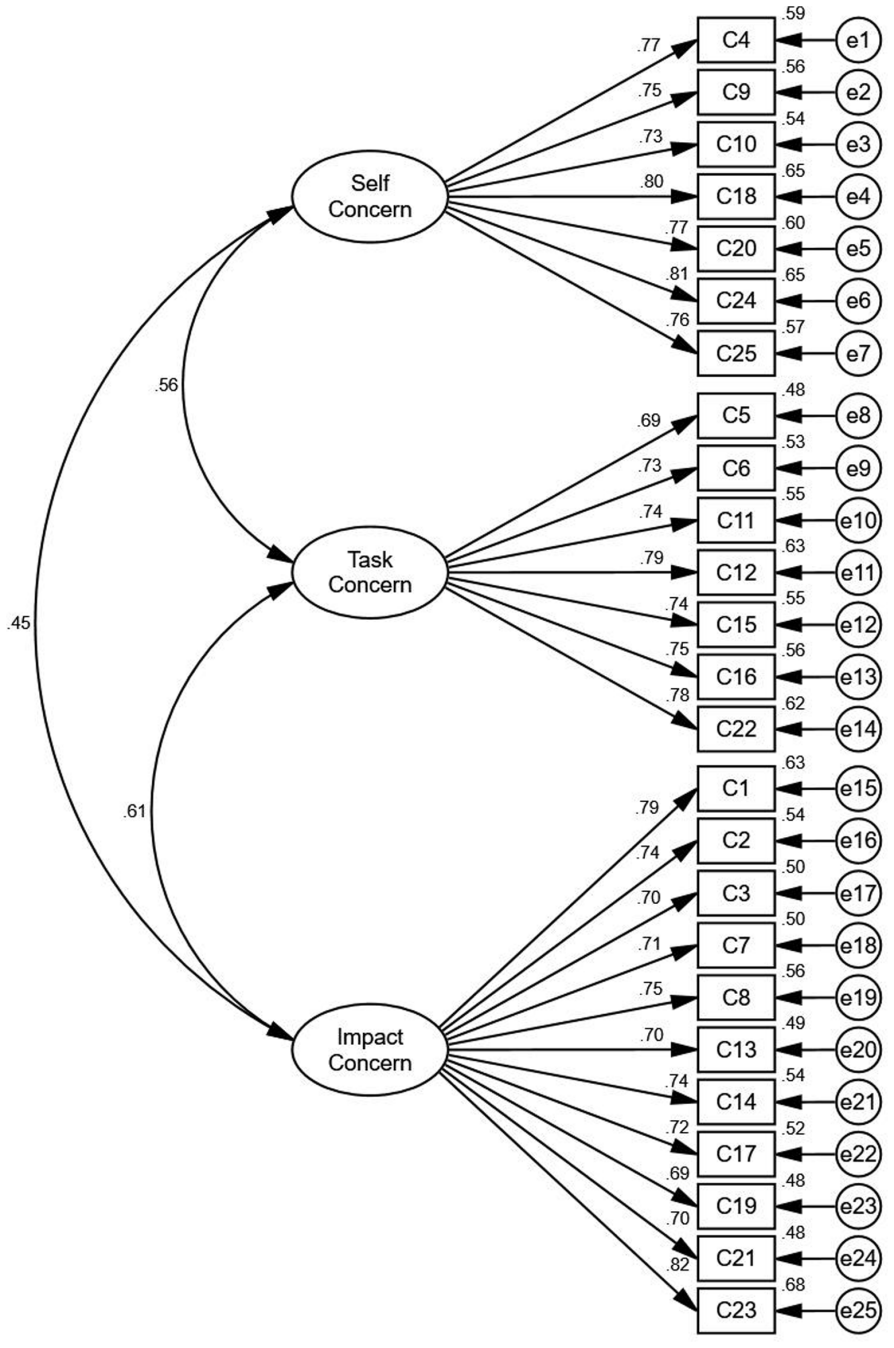
Confirmatory factor model (work concern).

**Figure 2:**
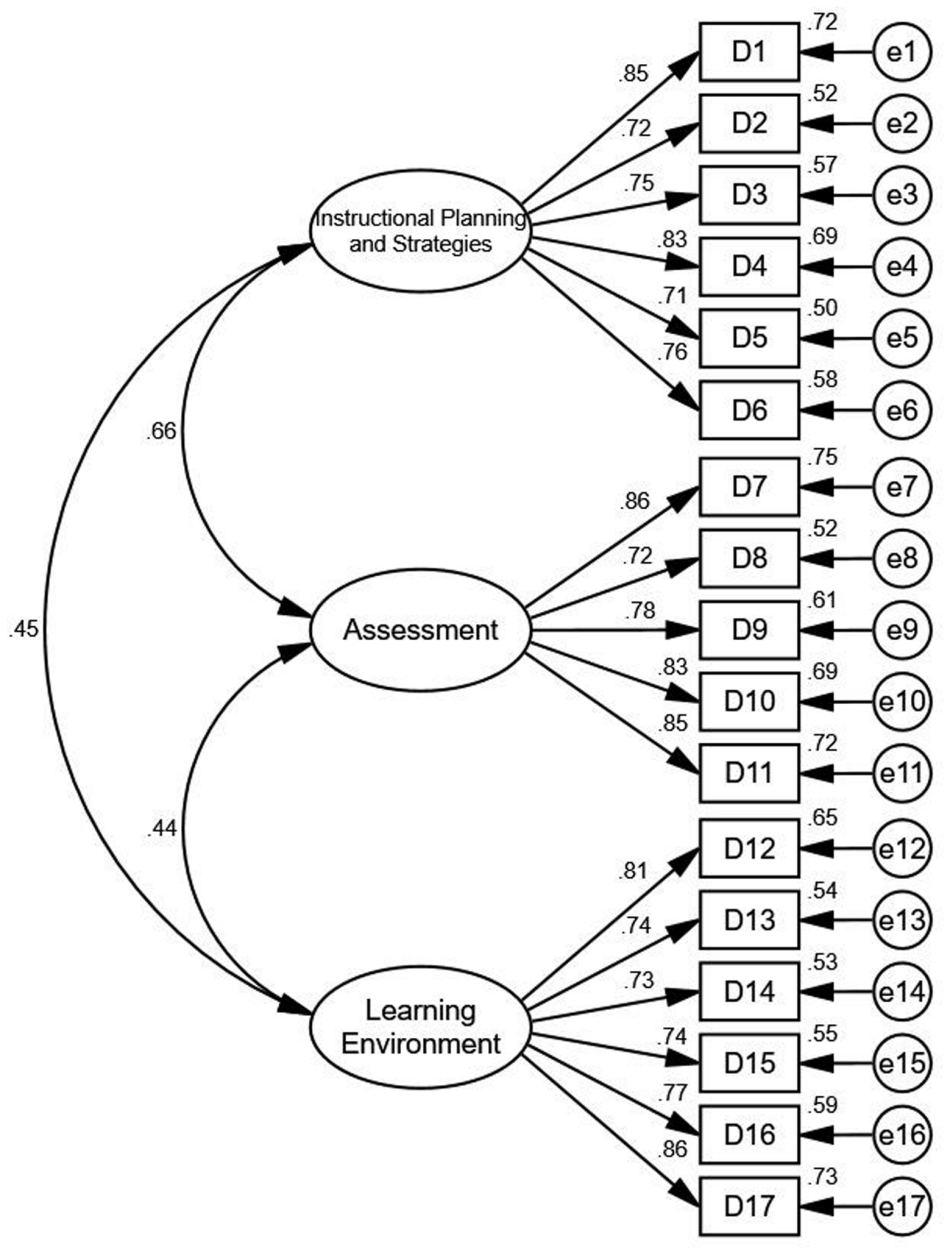
Confirmatory factor model (teacher effectiveness).

Confirmatory factor analysis (CFA) was also conducted to assess the model fit. The confirmatory factor models were shown in Figures 1, 2. Table 2 shows that X^2^/df values (1.425, 2.071) are all below 3, indicating acceptable model fit. GFI values (0.903, 0.913) and AGFI values (0.885, 0.886) are all above 0.8, meeting the required threshold. IFI values (0.972, 0.959) exceed 0.9, as do CFI and TLI, confirming good model fit. RMSEA values (0.038, 0.061) are all below 0.08, further validating the models. In addition, both the discriminant and convergent validity of the scales were confirmed, indicating that the measures were both reliable and valid for the purposes of the main study.

**Table 2:** Model fitting results.

Evaluating indicator	NC (X^2^/df)	GFI	AGFI	IFI	TLI	CFI	RMSEA
Work concerns	1.425	0.903	0.885	0.972	0.969	0.972	0.038
Teacher effectiveness	2.071	0.913	0.886	0.959	0.952	0.959	0.061
Adaptation standard	< 3	>0.8	>0.8	>0.9	>0.9	>0.9	< 0.08
Compliance with standards	Yes	Yes	Yes	Yes	Yes	Yes	Yes

### 2.5 Data analysis

The gathered data were analyzed through a combination of descriptive and inferential statistical methods. All responses were screened for completeness, accuracy, and consistency. After excluding incomplete or invalid entries, 416 valid questionnaires were retained for further analysis. The SPSS 26.0 and AMOS 24.0 were used for the data statistical analysis.

First, descriptive statistics were calculated to describe the background of the participants, including their gender, age, teaching experience, qualifications, and academic subject areas. The study looked at means, standard deviations, skewness, and kurtosis to check if the data were normally distributed (Hatem et al., 2022). To explore the connection between work concerns and teaching effectiveness, Pearson’s correlation was used, with a significance level set at 0.05. This method can help identify whether there were significant relationship between the different stages of work concerns and teaching effectiveness, as well as the strength and direction of these relationships (Schober et al., 2018).

Structural equation modeling (SEM) was also carried out to examine how work concerns influence teaching effectiveness. Path coefficients, standard errors, and *p*-values were reported to show the strength of these connections. In addition, multiple linear regression was conducted to further confirm how dimensions of work concerns could predict teaching effectiveness. In this analysis, the three stages of work concerns were used as predictors, while dimensions of teaching effectiveness were the outcome variables. The results included regression coefficients, R^2^ values, and p-values to see which factors had the strongest influence. Before running the regression, multicollinearity, homoscedasticity, linearity, and independence of residuals were test to ensure the results were reliable.

## 3 Results

### 3.1 Demographic characteristics

A total of 416 young full-time teachers participated in the study. Among them, 57.7% were female and 42.3% were male. In terms of age, the majority of respondents (39.4%) were between 25 and 30 years old, followed by 30–35 years (25.7%), 35–40 years (17.5%), and 25 and below (17.3%). Regarding teaching experience, more than half of the participants (55%) had between 1 and 5 years of experience. Additionally, 18.3% had 5–10 years of teaching experience, 19.2% had over 10 years, and 7.5% had one year or less. As for academic qualifications, most respondents held a master’s degree (78.4%), while 21.6% had obtained a doctoral degree. In terms of subject area, the largest group came from Arts, Social Sciences, and Humanities (29.8%), followed by Computing and Science (22.1%), Engineering and Technology (18%), Academic Subjects such as Chinese, English, and Mathematics (19%), Medicine and Pharmacy (8.4%), and others (2.6%).

### 3.2 Common method bias (CMB) test

In order to address the possible biases issue due to the reliance on self-reported measures, Harman’s single-factor test was conducted. The results of the exploratory factor analysis revealed that six factors had eigenvalues >1, and the first factor accounted for 36.230% of the total variance, which is below the recommended threshold of 40%. This indicates that common method bias is unlikely to pose a serious threat to the validity of the results (Baumgartner et al., 2021). To further strengthen the assessment, a CFA-based Common Latent Factor approach was applied, in which all measurement items were allowed to load on their respective constructs as well as on an unmeasured latent method factor. The analysis showed that the inclusion of the method factor did not substantially improve model fit, and the changes in the main path coefficients were all below 0.20. These findings suggest that common method bias does not pose a serious threat in this study.

### 3.3 Descriptive analysis

As presented in Table 3, the average score for overall work concerns is 3.567, suggesting that participants generally experience a moderate level of concern related to their professional roles. Among the three dimensions, Impact Concern shows the highest mean value (M = 3.651), indicating that respondents place greater emphasis on the influence their teaching has on others. In contrast, Task Concern records the lowest mean (M = 3.477), reflecting relatively less focus on day-to-day responsibilities. The standard deviations across the subscales indicate acceptable variability in responses. Additionally, all variables exhibited skewness and kurtosis within the acceptable −2 to +2 range, suggesting approximate normality of the data. This justifies the further use of parametric statistical techniques such as *t*-tests, correlation analysis, multiple regression, and structural equation modeling.

**Table 3:** Descriptive statistics result for the level of work concerns.

Components	Number of items	M	SD	Skewness	Kurtosis
Self-concern	7	3.573	0.938	−0.845	−0.277
Task Concern	7	3.477	0.878	−0.656	−0.422
Impact Concern	11	3.651	0.855	−0.658	−0.609
Overall	25	3.567	0.766	−1.033	0.324

Table 4 illustrates that the overall mean score of variable teaching effectiveness is 3.375, reflecting a moderate perception among the participants. Among the three dimensions, Instructional Planning and Strategies received the highest average rating (M = 3.527), suggesting that participants felt most confident in this area. In contrast, Assessment recorded the lowest mean score (M = 3.253), pointing to relatively lower perceived effectiveness in evaluating student learning. The standard deviations, ranging from 0.785 to 0.989, reflect a moderate degree of response dispersion across items. Moreover, the overall kurtosis value of −0.106 is very close to zero, implying that the distribution of responses approximates normality. Given the fulfillment of the normality assumption, the dataset is deemed suitable for conducting further parametric analyses, including independent samples *t*-tests, correlation, multiple regression, and structural equation modeling.

**Table 4:** Descriptive statistics result in the level of teaching effectiveness.

Components	Number of items	M	SD	Skewness	Kurtosis
Instructional planning and strategies	6	3.527	0.989	−0.513	−0.792
Assessment	5	3.253	0.948	−0.272	−1.017
Learning environment	6	3.345	0.879	−0.688	−0.200
Overall	17	3.375	0.785	−0.700	−0.106

The findings provide meaningful insights into the professional development dynamics of young university teachers. Overall, both work concerns and teaching effectiveness were found to be at moderate levels. Among the dimensions of work concerns, Impact Concern was rated highest, while Task Concern received the lowest score. These suggest that teachers who are early in their careers may be motivated by a desire to make a broader impact, even as they struggle with task-related responsibilities. Similarly, Instructional Planning and Strategies received the highest ratings within the teaching effectiveness scale, while Assessment was the lowest, possibly reflecting confidence in teaching preparation but uncertainties in evaluating student performance.

### 3.4 Multicollinearity test

In this study, multicollinearity diagnostics were performed to assess the independence of predictor variables. All tolerance statistics in Table 5 surpassed 0.1, and the corresponding VIF values did not exceed 10, ranging from 2.049 to 3.200. These results suggest that multicollinearity is not a concern in this study, supporting the reliability and stability of the regression analyses (Shrestha, 2020).

**Table 5:** Results of multicollinearity test.

Predictor variable	Tolerance	VIF
Self-concern	0.488	2.049
Task concern	0.313	3.200
Impact concern	0.441	2.270
Instructional planning and strategies	0.397	2.522
Assessment	0.369	2.708
Learning environment	0.334	2.991

### 3.5 Correlation analysis

The results of the correlation analysis in Table 6 indicate a strong, positive, and statistically significant relationship between overall work concerns and overall teaching effectiveness (r = 0.783, *p* < 0.01). At the component level, all three dimensions of work concerns (self-concern, task-concern, and impact-concern) were positively and significantly correlated with each aspect of teaching effectiveness. Among them, task concern showed the strongest correlation with the learning environment (r = 0.750, p < 0.01), while impact concern had the highest association with assessment (r = 0.569, p < 0.01).

**Table 6:** Correlation between work concerns and teaching effectiveness.

Work concerns component	Overall teaching effectiveness	Instructional planning and strategies	Assessment	Learning environment
Overall work concerns	0.783**	–	–	–
Self-concern	–	0.477**	0.513**	0.543**
Task concern	–	0.559**	0.547**	0.750**
Impact concern	–	0.546**	0.569**	0.605**

**Correlation is significant at the 0.01 level (2-tailed).

These results indicate that higher level of work concerns, especially task and impact concerns, are more correlated with effective teaching practices such as planning, assessment, and classroom environment. This means that teachers who pay more attention to their daily tasks and school expectations are more likely to build a supportive learning environments. This supports earlier research that highlights how being clear about one’s role and staying aware of daily responsibilities can lead to better teaching (Almarode and Vandas, 2018; Roksa et al., 2017). On the other hand, the correlation between self-concern and teacher effectiveness is weaker.

### 3.6 Structural equation model (SEM)

**Figure 3:**
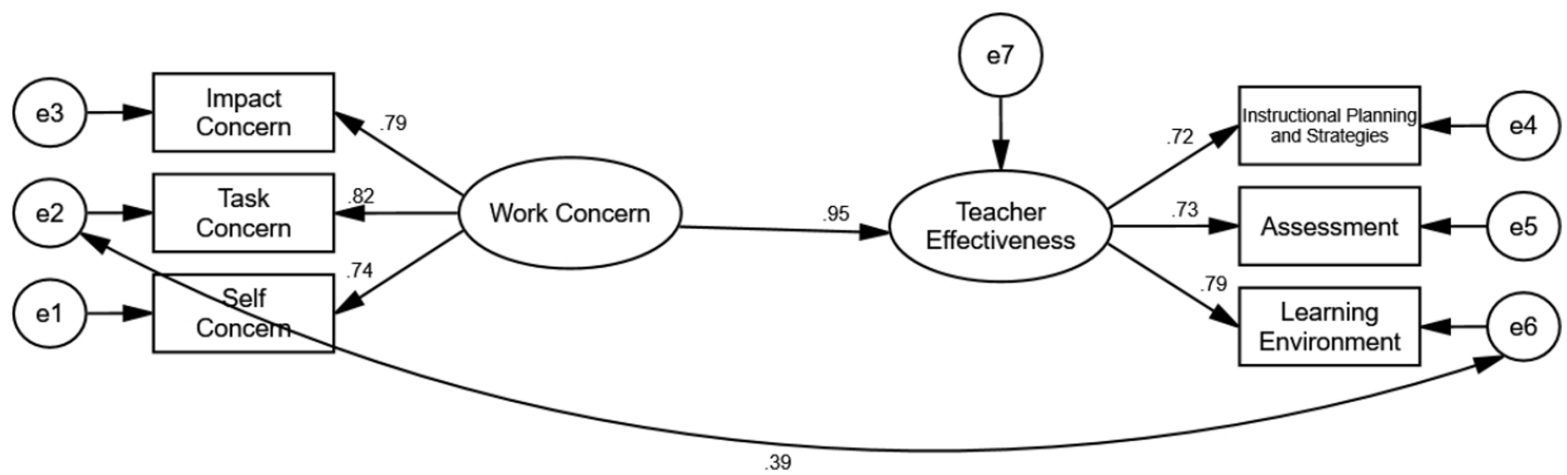
Structural equation model (SEM).

The structural equation model in Figure 3 illustrates the direct relationships among the latent constructs of Work Concern and teaching effectiveness, along with their respective observed variables. The three dimensions: Self-concern (*β* = 0.74), Task Concern (*β* = 0.82), and Impact Concern (*β* = 0.79), which load strongly onto the latent variable Work Concern, indicating that all three are significant contributors to this construct. Work Concern shows a very strong direct effect on teaching effectiveness (*β* = 0.95), suggesting that teachers who express higher concern levels related to their work also tend to perceive themselves as more effective in their roles. Teaching effectiveness is further reflected by three components: Instructional Planning and Strategies (*β* = 0.72), Assessment (*β* = 0.73), and Learning Environment (*β* = 0.79), all of which show strong standardized loadings, confirming that these dimensions reliably represent the overall construct. Additionally, a moderate positive correlation (r = 0.39) exists between the latent variables of Work Concern and teaching effectiveness, further supporting the positive association between these two key constructs.

The structural equation model demonstrated an excellent fit to the data shown in Table 7. The chi-square statistic was non-significant (χ^2^ = 9.316, df = 7, *p* = 0.231), and the ratio χ^2^/df = 1.331 fell well below the recommended threshold of 3. Goodness-of-fit indices were all above conventional cutoffs (GFI = 0.992, AGFI = 0.977, NFI = 0.993, IFI = 0.998, TLI = 0.996, CFI = 0.998), indicating strong model performance. The residual-based indices also supported a good fit, with RMSEA = 0.028 (90% CI [0.000, 0.071], PCLOSE = 0.759) and SRMR = 0.011, both within acceptable limits. Collectively, these results confirm that the proposed model provides an excellent representation of the observed data.

**Table 7:** Structural equation model (SEM) fit.

Index	X^2^/df	GFI	AGFI	NFI	IFI	TLI	CFI	RMSEA	SRMR
Statistical value	1.331	0.992	0.977	0.993	0.998	0.996	0.998	0.028	0.011
Reference value	<5	>0.8	>0.8	>0.9	>0.9	>0.9	>0.9	<0.08	<0.08
Fit achieved	Yes	Yes	Yes	Yes	Yes	Yes	Yes	Yes	Yes

The SEM analysis showed a good model fit and confirmed the hypothesized structural relationships. In this model, we can find that the three dimensions of work concern (self-concern, task-concern, and impact-concern) have a strong direct effect on teaching effectiveness. Each of the dimensions of teaching effectiveness (instructional planning and strategies, assessment, and learning environment) also showed strong loadings. These findings provide empirical validation of the conceptual framework. These results are similar to transformational and concern-based perspectives on professional growth (Kyndt et al., 2016; Toropova et al., 2021; Wartenberg et al., 2023). Nwoko et al. (2023) underscored the significance of work concerns in the private university sector, where job stability and workload concerns are prevalent.

### 3.7 Multiple regression analysis

The multiple linear regression model in Table 8 examined the influence of work concerns dimensions and demographic variables on teaching effectiveness across three components. The results showed that task concern and impact concern were consistently strong and significant predictors across all three domains: instructional planning and strategies, assessment, and the learning environment (*p* < 0.001). Specifically, task concern demonstrated the highest standardized beta (*β* = 0.568, t = 12.451***) in predicting the learning environment, highlighting its critical role. In contrast, self-concern showed a weaker and only marginally significant effect on instructional planning (*β* = 0.105, t = 2.026*) and assessment (*β* = 0.178, t = 3.471**), and a non-significant effect on the learning environment (*β* = 0.042, t = 0.982). Among demographic variables, working experience significantly influenced all three components, while gender showed a significant effect only on instructional planning. The models explained 39.4 to 59.8% of the variance in the dependent variables, with the highest explanatory power observed for the learning environment (Adjusted R^2^ = 0.598, *F* = 78.326***).

**Table 8:** Multiple linear regression analysis for work concerns on teaching effectiveness.

Predictor variables	Instructional planning and strategies		Assessment		Learning environment	
	*β*	t	*β*	t	*β*	t
Gender	0.089	2.292*	0.003	0.07	0.057	1.794
Age	0.012	0.264	0.036	0.798	0.003	0.069
Working experience	0.112	2.427*	0.112	2.466*	0.088	2.355*
Qualification	0.01	0.256	−0.003	−0.071	0.049	1.537
Subject area	0.04	1.047	0.046	1.208	0.048	1.55
Self-concern	0.105	2.026*	0.178	3.471**	0.042	0.982
Task concern	0.274	4.895***	0.196	3.543***	0.568	12.451***
Impact concern	0.274	5.319***	0.317	6.233***	0.193	4.595***
R^2^	0.405		0.421		0.606	
Adjusted R^2^	0.394		0.409		0.598	
F	34.687***		36.938***		78.326***	

*P < 0.05, **p < 0.01, ***p < 0.001.

The results of multiple regression further confirmed the predictive capacity of work concerns on teaching effectiveness. All three types of concern were significant predictors, with task concern emerging as the most consistent and influential factor across all dimensions of teaching effectiveness. This underscores the practical implication that helping young teachers develop task-related competencies, such as managing time, materials, and expectations, could substantially enhance their effectiveness. Impact concern also significantly predicted all aspects of effectiveness, reinforcing the idea that teachers who emphasize the impact of their work are more engaged in evaluating student progress and creating meaningful learning experiences. They actively seek ways to improve their teaching effectiveness, making them more adaptable to student needs. Contrary to some earlier assumptions, self-concern showed weaker and less consistent effects. Although it showed a significant predictive effect on instructional planning and assessment, it had no notable impact on the learning environment. This implies that personal anxieties or uncertainties may be less directly impact on how teachers manage their classrooms. The study confirms that work concerns are not simply emotional burdens and challenges but also key indicators and even possibly drivers of teaching effectiveness. In addition, The regression analysis shows that work concerns have a significant impact on teacher effectiveness (R^2^ = 0.405–0.606, *p* < 0.001), explaining between 40 and 60% of the variation of teacher effectiveness. However, a portion of the variance remains unexplained.

## 4 Discussion

### 4.1 Level of work concerns of young teachers in Shandong’s private universities

The results show that young teachers in Shandong’s private universities generally have a moderate level of work concerns. They are aware of the challenges in their jobs, but these concerns are not overly negative. Among the different types, impact concern was the highest, which means they care deeply about how their work influences students’ learning and future success. This supports Day (2013) who found that university teachers often see themselves as playing an important role in shaping student outcomes. At the same time, self-concern was also quite strong, while task concern scored the lowest. This may be because many young teachers in private universities face job instability and limited promotion opportunities compared to those in public universities (Gu and Zhang, 2023). These conditions likely make them more focused on their personal career development and long-term stability.

These findings are consistent with earlier studies showing that early-career teachers tend to worry about job security and their impact on student success (Skaalvik and Skaalvik, 2017; Yang, 2023). The results also fit Fuller’s (1969) Concern-Based Adoption Model (CBAM), which explains how teachers’ concerns usually shift from personal issues to teaching tasks, and finally to student outcomes. Interestingly, young teachers in this study already show a strong sense of responsibility for student learning, which may reflect their commitment to their work despite facing career uncertainties. To better support them, universities should offer clearer promotion pathways, professional development programs, and mentoring opportunities (Darling-Hammond, 2017).

### 4.2 Level of teaching effectiveness of young teachers in Shandong’s private universities

The study found that young teachers in Shandong’s private universities generally show a moderate level of teaching effectiveness. Among the three dimensions, Instructional Planning and Strategies scored the highest, followed by Learning Environment, while Assessment was the lowest. This suggests that most young teachers are confident in their ability to plan lessons and manage classroom, which is often emphasized in teacher training (Darling-Hammond, 2017). However, they seem not skilled when it comes to student assessment because it requires deeper understanding and skills in both formative and summative evaluation (Black and Wiliam, 2018). These moderate results may also reflect the tough working conditions in private universities, where young teachers often face heavy workloads, short-term contracts, and fewer development opportunities compared to their peers in public universities (Liu, 2023).

These findings match previous studies showing that teacher effectiveness can vary in different dimensions and is often influenced by the work environment (Gu and Zhang, 2023; Stronge, 2018). Previous research in Chinese higher education also noted that young private university teachers tend to perform well in instructional strategies but often struggle with assessment and student engagement (Wen-ying and Xi, 2016). According to Bandura and Wessels (1994), teachers who have higher confidence are more likely to try new approaches and improve their teaching. For many young teachers, building self-efficacy and improving teaching effectiveness takes time and experience. Therefore, universities should provide targeted support, such as assessment training workshops, peer learning, and feedback systems, to help young teachers grow in areas where they feel less confident, especially in student assessment practices.

### 4.3 Relationship between work concerns and teaching effectiveness

The findings of this study show that teachers’ work concerns are closely related to their teaching effectiveness. Among the different stages of concerns, task concern had the strongest positive relationship with teaching effectiveness. This means that when teachers actively work on improving their lessons, getting students involved, and refining their teaching methods, they are likely to perform better (König et al., 2024). Impact concern also showed a strong connection to effectiveness. Teachers who care about student success often seem more motivated and tend to teach better (Liu, 2024). These results align with earlier studies that teachers who see their work as meaningful are often more committed and effective (Buhl-Wiggers et al., 2017; Kumar, 2023). On the other hand, self-concern had a weaker but still positive link to teaching effectiveness. Teachers who are mainly focused on personal concerns might not put as much energy into improving their teaching as those who are focused on student success (Zhang et al., 2024).

These results suggest that teachers in private universities seem to care more about improving their teaching and helping students than worrying about job security. This finding is different from some past studies, such as Gómez-Domínguez et al. (2023), who reported that job insecurity can lead to lower teacher engagement. In private universities, teachers seem more driven by their teaching responsibilities. Harrison et al. (2023) also pointed out that teachers who put more effort into improving their teaching and assessment skills tend to feel more satisfied and effective. For private universities, this means creating a supportive work environment really matters. Leaders should offer clear career paths, reasonable workloads, and professional development opportunities to help reduce self-concerns. At the same time, leaders should strengthen task and impact concerns through mentorship, peer collaboration, and regular feedback because it can help teachers stay focused on improving their teaching. Building a culture where teachers feel their work could be valued is important too because it helps to improve teaching quality and student outcomes.

### 4.4 Predictive influence of work concerns on teaching effectiveness

Task concern had the strongest influence on teaching effectiveness, particularly in shaping the learning environment dimension. Impact concern also showed a strong positive effect, especially in assessment dimension. It means that teachers who are more concerned about their tasks and the impact of their teaching tend to be more effective, especially in creating supportive learning environments and improving student assessment practices. In contrast, self-concern had a weaker influence and was not significantly related to the learning environment dimension of teaching effectiveness. These findings are consistent with previous studies (Boz and Cetin-Dindar, 2023; Gul et al., 2021; Sinclair, 2024), which suggest that teachers who concentrate on improving instruction and making a good learning environment are more effective in the classroom. This also supports Fuller’s (1969) teacher development model, which indicates that early-career teachers often focus on task concerns because they can build their teaching confidence.

The weaker influence of self-concern compared to task and impact concerns may be partly explained by cultural and institutional factors in Chinese private universities. In China’s collectivist context, teachers’ professional identity is shaped more by external expectations—student results, institutional demands, and peer evaluations—than by personal anxieties. In private universities, appraisal and promotion are also closely tied to student feedback and measurable outcomes, which leads teachers to prioritize tasks and impact over self-focused concerns. Practically, interventions should align with the areas where concerns most strongly predicted effectiveness. Since task concerns were most influential for the learning environment (R^2^ = 0.606), support such as fair workload distribution and classroom resources is essential. Because impact concerns were tied to assessment, mentorship can focus on helping young teachers use assessment not just for grading but to enhance learning. These implications are directly linked to the observed results.

The regression results further show that work concerns can explain around 40 to 60% of the variation in teaching effectiveness across different dimensions. It means that teachers’ perceptions of their work, such as support, pressure, and their influence on students, play a key role in how they manage the classroom. However, not all teaching effectiveness can be explained by work concerns alone. There could be some other factors like school culture, leadership support, and collegial relationships also play an important role in teaching effectiveness (Gibbons, 2023; Picaza et al., 2022). Future research may explore how other factors, such as institutional policies, leadership support, or teacher motivation, interact with work concerns to influence teacher effectiveness over time.

## 5 Conclusion

This study investigates how work concerns shape professional growth by exploring their impact on teaching effectiveness among young lecturers in Chinese private universities. The findings show that task-concerns and impact-concerns have a much stronger influence on teaching effectiveness, while self-concerns have only a small effect. These results suggest that leaders in private universities should pay more attention to the work-related needs of young teachers because it can help to improve their teaching performance. This study provides empirical evidence that links work concerns to teaching effectiveness and gives a better understanding of teacher development in China’s private university. There are also limitations in this study. The cross-sectional design and sample in only one province limit the generalizability of the results. Future research could consider longitudinal and multi-site studies that can validate and extend the findings.

## Data Availability

The raw data supporting the conclusions of this article will be made available by the authors, without undue reservation.
